# Transportation assimilation revisited: New evidence from repeated cross-sectional survey data

**DOI:** 10.1371/journal.pone.0194296

**Published:** 2018-04-18

**Authors:** Dafeng Xu

**Affiliations:** Minnesota Population Center, University of Minnesota, Minneapolis, MN 55414, United States of America; Beijing Jiaotong University, CHINA

## Abstract

**Background:**

Based on single cross-sectional data, prior research finds evidence of “transportation assimilation” among U.S. immigrants: the length of stay in the U.S. is negatively correlated with public transit use. This paper revisits this question by using repeated cross-sectional data, and examines the trend of transportation assimilation over time.

**Methods and results:**

Using 1980, 1990, 2000 1% census and 2010 (1%) American Community Survey, I examine the relationship between the length of stay in the U.S. and public transit ridership among immigrants. I first run regressions separately in four data sets: I regress public transit ridership on the length of stay, controlling for other individual and geographic variables. I then compare the magnitudes of the relationship in four regressions. To study how the rate of transportation assimilation changes over time, I pool the data set and regress public transit ridership on the length of stay and its interactions with year dummies to compare the coefficients across surveys. Results confirm the conclusion of transportation assimilation: as the length of stay in the U.S. increases, an immigrant’s public transit use decreases. However, the repeated cross-section analysis suggests the assimilation rate has been decreasing in the past few decades.

**Conclusions:**

This paper finds evidence of transportation assimilation: immigrants become less likely to ride public transit as the length of stay in the U.S. increases. The assimilation rate, however, has been decreasing over time. This paper finds that the rate of public transit ridership among new immigrants upon arrival, the geographic distribution of immigrants, and the changing demographics of the U.S. immigrants play roles in affecting the trend of transportation assimilation.

## Introduction

Compared with native-born commuters, U.S. immigrants generally have different transportation patterns: they are more likely to ride public transit and less likely to drive [1–5]. This is especially true when immigrants just arrive in the U.S.: many of them have low socioeconomic status, have no driver’s license that can be used in the U.S., and are less familiar with local road systems. After arrival, however, immigrants gradually become socially [6, 7], economically [8, 9], and culturally assimilated [10, 11] into the U.S. society. As a result, they might also assimilate into the transportation pattern in the U.S. by taking less public transit.

Following the assimilation framework [12], prior transportation research [13] finds that transportation assimilation does occur among U.S. immigrants in California: when immigrants stay in the U.S. longer, they are less likely to take public transit to commute. The conclusion of transportation assimilation is robust even after controlling for demographic and socioeconomic variables. This paper extends prior research along two dimensions. First, I study immigrants living in the entire U.S. rather than a state. Second, and more importantly, I use repeated cross-sectional census and American Community Survey (ACS) data taken in 1980, 1990, 2000, and 2010, rather than a single cross-sectional data set. By using survey data taken in different years, this paper takes the possible systematic differences between various cohorts of immigrants into account: using single and repeated cross-sectional data to study transportation assimilation are equivalent only if the immigration flow is constant over time and immigrants of different cohorts respond to the U.S. transportation systems in the same way. Therefore, it is important to revisit the empirical question of transportation assimilation among U.S. immigrants using new data sets, in particular, repeated cross-sectional data.

### Theoretical framework

From immigrants’ perspective, there are mainly three types of determinants of transportation mode choices. First, various demographic and socioeconomic characteristics affect choices of transportation modes. Automobile use is more costly than public transit use, hence all else being equal, socioeconomic status should be negatively correlated with public transit use [14, 15]. Recent studies suggest that other socioeconomic variables, such as education [16], are also related to transportation mode choices. Second, geographic factors affect choices of transportation modes. In general, larger cities provide better public transit services and also have problems of traffic congestion, while in many rural areas there are even no public transit systems at all [17]. That said, this geographic pattern is highly heterogeneous in the U.S.: cities with similar population sizes might have very different public transit systems, depending on their specific urban forms and policies [1, 17–20]. Third, culture also determines choices of transportation modes. The public transit ridership rate is much lower in the U.S., partially due to the cultural preference of automobile use [18].

The above points explain why U.S. immigrants have different transportation patterns, i.e., the higher propensity of public transit ridership. First, immigrants, especially those with shorter length of stay, might have lower socioeconomic status [8]. Second, there is a striking difference in the geographic distribution of the native-born and immigrant population in the U.S. [6, 7], especially in recent decades [21–23]. Finally, many immigrants encounter cultural conflicts and do not accept the American culture immediately after arrival. The above arguments, however, also indicate that immigrants should assimilate into the “regular” transportation pattern in the U.S. as the length of stay increases. Economists and sociologists have long documented that immigrants economically [8, 9], geographically [6], and culturally [7] assimilate into the U.S. society. In particular, when staying long enough in the U.S., immigrants and natives generally have similar socioeconomic status and settlement patterns.

In the statistical analysis, scholars can include various socioeconomic and geographic characteristics as covariates and thus capture the contributions of economic and geographic assimilation to transportation assimilation. Year fixed effects and the interaction between year and geographic characteristics should also be controlled as there might be time-specific regional factors (e.g., recent development of public transit systems) that affect public transit ridership. However, even after controlling for these variables, the length of stay in the U.S. might still be related to choices of transportation modes. In general, sociocultural characteristics are not surveyed and are thus omitted in most data sets, yet the length of stay in the U.S. still affects social and cultural assimilation [7], which could further affect choices of transportation modes: for example, all else being equal, an immigrant who stays in the U.S. longer might be more willing to accept the driving culture in the U.S., or more familiar with the highway system in the U.S., and thus more likely to drive and less likely to ride public transit. Such social and cultural mechanisms should be reflected in the relationship between the length of residency in the U.S. and choices of transportation modes, conditional on other individual and geographic characteristics [13].

### Prior literature on immigrant cohorts

Following the above framework, although there might be a consistent qualitative relationship between the length of stay in the U.S. and the degree of similarity between immigrants’ and natives’ behavior (in this paper, public transit ridership), the rate of assimilation could be cohort-specific. Here I briefly discuss prior studies that compare social assimilation of U.S. immigrants of different cohorts, which helps understand not only how, but also why the trend of transportation assimilation could change over time.

Many scholars focus on differences in individual characteristics between different cohorts of immigrants and relate such differences to immigrants’ social outcomes. For example, cohort trends of residential crowding among immigrants are very different from the cross-sectional pattern [24]. Related to transportation, while it is correct that recent immigrants are more likely to take public transit (hence less “assimilated”) [5, 13], immigrants of different cohorts have different individual characteristics [3, 25], and thus could assimilate to the “social transportation norm” at different rates [26]. Specifically, the age and racial composition of U.S. immigrants have greatly changed in recent decades. U.S. censuses suggest the average length of stay among immigrants has increased over the past decades [27]. Furthermore, the U.S. has experienced the severe downturn of European immigrants [27]. While there was once a significant increase in Hispanic immigrant populations (especially those from Mexico), there is actually a declining trend of Hispanic immigration in recent years [28]. On the other hand, the Asian population in the U.S. has steadily increased since the 1970s [27] after the new immigration policies were adopted and many Asian people started to move to the U.S. These suggest that the U.S. immigrant population of different cohorts should have different demographic characteristics.

Furthermore, immigrants of different cohorts have different settlement patterns and face changes in the transportation system over time. Traditionally, immigrants are more likely to live in urban areas and prefer ethnic enclave residence [6, 7, 12, 21], but many new immigrants tend to choose new locations, such as areas that were previously unfavored destinations [22, 23] and suburban areas (i.e., “ethnoburbs” [29, 30]). Moreover, immigrants of different cohorts could experience different U.S. transportation systems. In the context of this paper, driving is significantly affected by oil price shocks in the late 20th century [31]. In recent decades, public transit systems in the U.S.—especially in the West—also affect travel behaviors of commuters [32, 33], including immigrants. As suggested in the theoretical framework, year fixed effects and geographic variables are needed to be included in the empirical analysis in order to control for these factors.

### Contributions

Understanding transportation assimilation has important policy implications. As mentioned earlier, immigrants constitute a major part of public transit riders in the U.S.; in some areas, the proportion of foreign-born transit commuters can be as high as 50% [3]. Researchers rely on the empirical analysis of transportation assimilation to predict the future of transit ridership [5, 25]. The study of transportation assimilation based on multiple historical data sets could provide more accurate estimation for future public transit because it takes immigration-driven population dynamics in the U.S. into consideration [3, 26].

Studies of transportation assimilation among U.S. immigrants also shed light on transportation policies in terms of facilities and services for immigrant passengers [3]. If immigrants are still the large population of public transit riders in the future, then it is useful to provide facilities and services for the rising immigrant population in the public transit system, such as multilingual signs and announcements. So far, a few cities (e.g., New York and San Francisco) do have provided such services as these cities have long been immigrants’ favored destinations, and many immigrants use public transit after settling down. This could be employed in the entire country if other cities similarly experience the rapid growth of the immigrant transit market.

## Materials and methods

### Data

To study transportation assimilation in 1980, 1990, and 2000, this paper uses Integrated Public Use Microdata Series (IPMUS) 1980, 1990, and 2000 1% census data [27]. For 2010, I use the IPUMS American Community Survey (ACS) that similarly contains 1% of the nationally representative population and asks the same questions as previous censuses [27] (questions in the long form census were no longer asked in the 2010 census; instead, they were transferred to the ACS).

The U.S. census and ACS survey respondents’ transportation tools for commuting to work. These data sets provide information about the residence and workplace. Hence, The sample only contains individuals who are employed. I also exclude those who work from home as they need not to commute. These suggest one limitation of this paper: as the surveys only ask questions about transportation modes for commuting, I cannot study immigrant’s transportation behaviors for other purposes, such as shopping [13].

The total sample size is 4,553,519, in which 941,617 are from the 1980 1% census, 1,100,783 are from the 1980 1% census, 1,227,865 are from the 2000 1% census, and 1,283,254 are from the 2010 ACS. I only use the immigrant sample in this study: among all individuals in the sample, 537,566 are foreign-born individuals who immigrated from other countries. 62,934 are from the 1980 1% census, 106,740 are from the 1990 1% census, 167,443 are from the 2000 1% census, and 200,449 are from the 2010 ACS.

### Variables

The dependent variable in the statistical analysis is a binary indicator of public transit ridership. Although transportation assimilation indicates both the lower likelihood of public transit use and higher likelihood of automobile use, in this paper I do not construct an automobile use indicator because it is highly correlated with public transit ridership: besides drivers and public transit riders, only approximately 5% of all commuters in the sample choose other transportation modes. Note that this paper focuses specifically on immigrants’ public transit ridership, but do not exclude transportation modes other than automobile use.

The length of stay in the U.S. is the key independent variable of the main interest. As discussed earlier, there are mainly three types of variables related to transportation assimilation other than the length of stay. First, transportation mode choices are affected by individuals’ demographic and socioeconomic characteristics, including gender, age, race and ethnicity, marital status, country of origin, years of schooling, and income. Table 1 presents descriptive statistics of these variables. Second, geographic factors affect transportation mode choices. All else being equal, a person living in an area with better public transit systems are more likely to take public transit. I include county-year fixed effects that control for all observable and unobservable time-variant area-specific factors. Finally, social and cultural assimilation affect transportation assimilation. However, surveys generally do not ask related questions.

**Table 1:** Descriptive statistics: The immigrant sample.

	Full	1980	1990	2000	2010
	sample	census	census	census	ACS
Public transit	0.114	0.156	0.124	0.112	0.096
	(0.317)	(0.363)	(0.330)	(0.315)	(0.295)
Length of stay in the U.S	17.455	12.794	14.841	17,084	20.621
	(12.437)	(9.907)	(10.795)	(12.363)	(13.217)
Female	0.423	0.410	0.413	0.413	0.441
	(0.494)	(0.492)	(0.492)	(0.492)	(0.496)
Age	39.862	39.077	37.805	38.468	42.369
	(12.779)	(13.735)	(12.645)	(12.366)	(12.468)
Working in central metropolitan areas	0.248	0.243	0.365	0.226	0.205
	(0.432)	(0.429)	(0.482)	(0.418)	(0.404)
Working outside metropolitan areas	0.052	0.028	0.060	0.050	0.056
	(0.221)	(0.167)	(0.237)	(0.217)	(0.230)
Hispanic, white	0.238	0.295	0.205	0.193	0.274
	(0.426)	(0.456)	(0.404)	(0.394)	(0.446)
Hispanic, others	0.180	0.030	0.211	0.238	0.161
	(0.384)	(0.169)	(0.408)	(0.426)	(0.368)
Non-hispanic white	0.264	0.446	0.306	0.234	0.209
	(0.441)	(0.497)	(0.461)	(0.423)	(0.407)
Black	0.073	0.065	0.066	0.074	0.079
	(0.261)	(0.246)	(0.248)	(0.262)	(0.270)
Asian	0.237	0.174	0.226	0.234	0.267
	(0.426)	(0.380)	(0.418)	(0.423)	(0.442)
Married	0.640	0.686	0.635	0.620	0.645
	(0.480)	(0.464)	(0.497)	(0.485)	(0.478)
Number of children	1.094	1.136	1.094	1.065	1.106
	(1.289)	(1.393)	(1.321)	(1.288)	(1.236)
Years of schooling	12.364	11.567	11.885	12.364	12.869
	(4.377)	(4.473)	(4.553)	(4.291)	(4.256)
Personal income (2010 $)	41929.49	36775.61	38381.90	42067.39	45327.83
	(51603.84)	(34204.83)	(44314.97)	(56040.84)	(55483.20)
Observations	537,566	62,934	106,740	167,443	200,449

Standard deviations are in parentheses.

In Table 1, I find that 11.4% of all immigrants in the sample choose public transit as the commuting tool. In a sharp contrast (which is not reported in this table), only 4% of native-born commuters take public transit. The rate of public transit ridership among immigrant commuters decreases over time—15.6% in the 1980 census, and only 9.6% in the 2010 ACS. This might be closely related to the trend of length of stay in the U.S. over time: the average length of stay is 17.5 years in the sample, but the length of stay in the U.S. among immigrants has significantly increased over time, from 12.8 years in 1980 and 20.6 years in 2010. Note that options for the question of the length of stay were not given as exact years; only year intervals (e.g., 1 to 5 years) in the 1980 and 1990 census were surveyed. Hence, I am only able to assign the average length of stay in the year interval to individual immigrants in the sample. However, given these intervals are fairly small (within several years), I assume that the within-interval length of stay follows some symmetric distribution (e.g., uniform distribution), and thus measurement error should not lead to systematically biased estimates of transportation assimilation.

The place of work plays a crucial role in determining travel behaviors. Some central cities provide good public transit systems; moreover, parking and congestion could be difficult for drivers. On the other hand, many non-metropolitan areas do not have any public transit system at all. 24.8% of immigrants work in central parts of metropolitan areas, and 5.2% of immigrants work outside metropolitan areas.

42.3% of immigrant commuters are female. There are more female commuters in 2010, possibly due to the increasing female employment rate. The average age in the sample is close to 40 years old. 42% of all immigrant commuters are Hispanic, and Table 1 shows the mass influx of Hispanic immigrants after 1980, although the proportion of Hispanic immigrants has gradually become stable after 2000. More than half of Hispanic immigrants self-identify as white. 26.4% of immigrants are non-Hispanic whites, and 7.3% are black; the proportion of non-Hispanic white immigrants has largely decreased since 1980. On the contrary, the proportion of Asian immigrants has increased since 1980. The marriage rate in the sample is fairly stable: approximately 64% of all immigrant commuters are married. The average number of children is about 1. Finally, the average years of schooling are 12.4 years, and the average personal income (in 2010 USD) is 42,000 dollars. Both years of schooling and personal income have increased over time among immigrant commuters.

**Fig 1:**
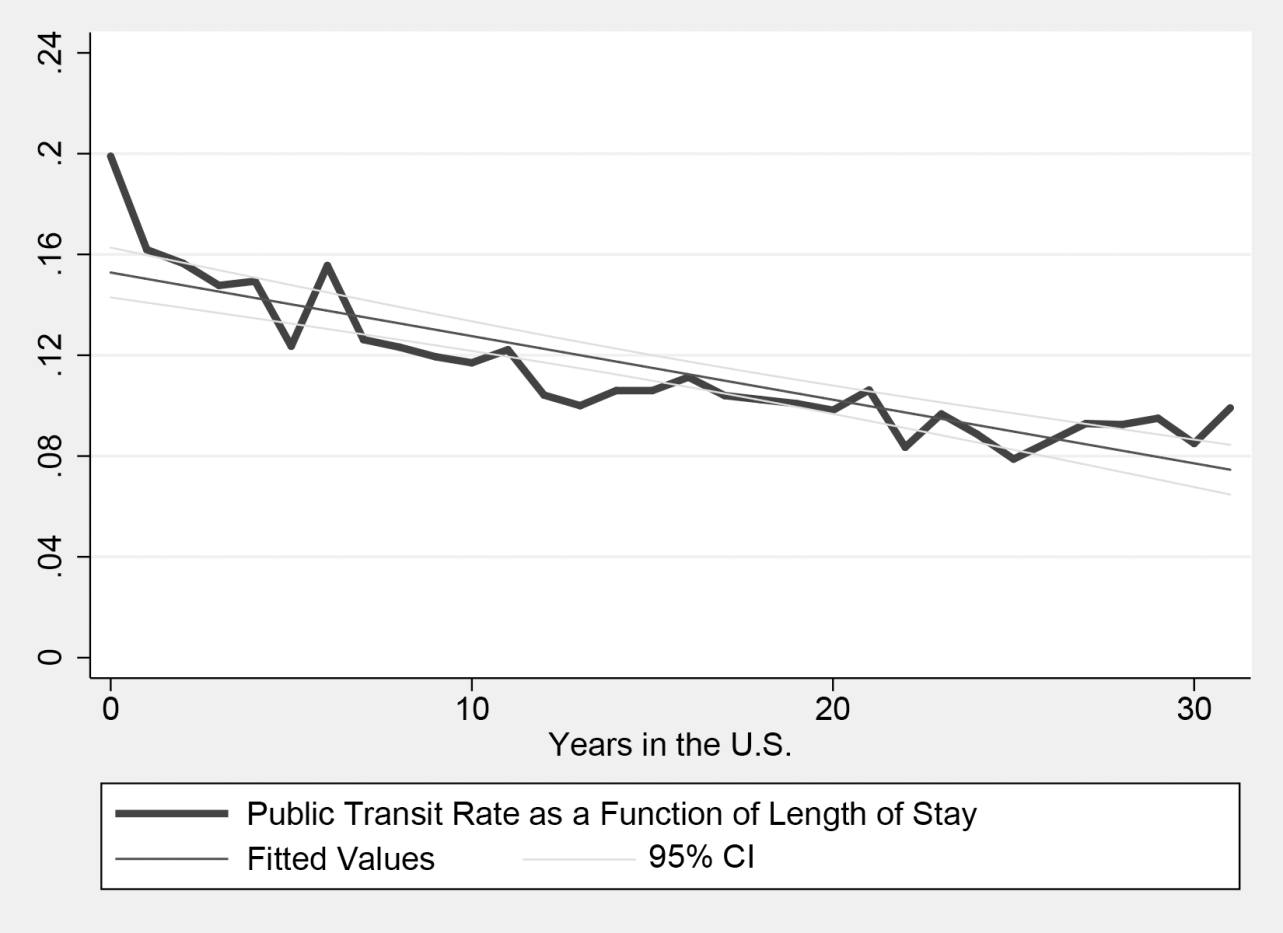
Public transit rate and the length of stay: Full (1980—2010) sample.

**Fig 2:**
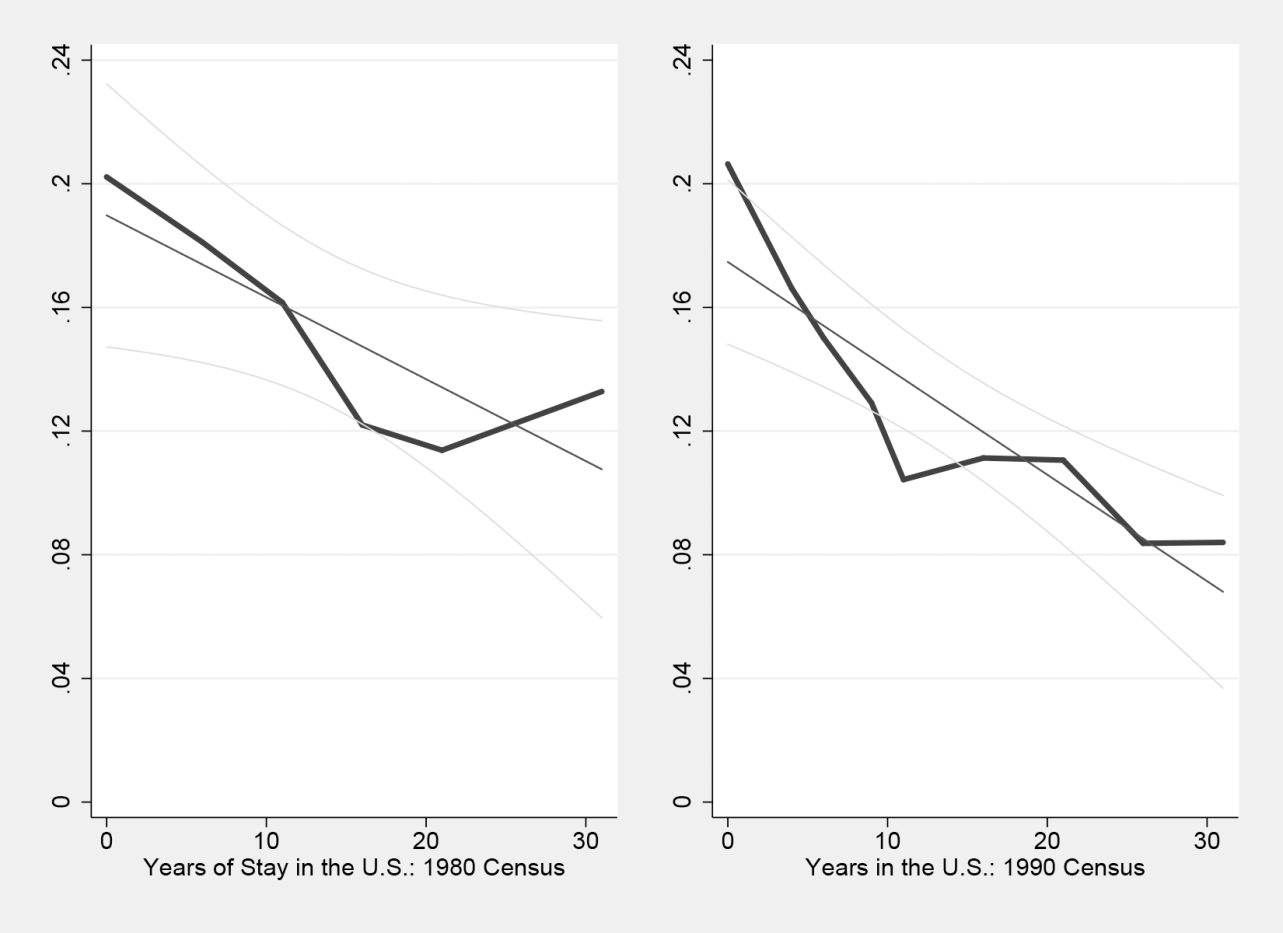
Public transit rate and the length of stay: Prior to 2000.

**Fig 3:**
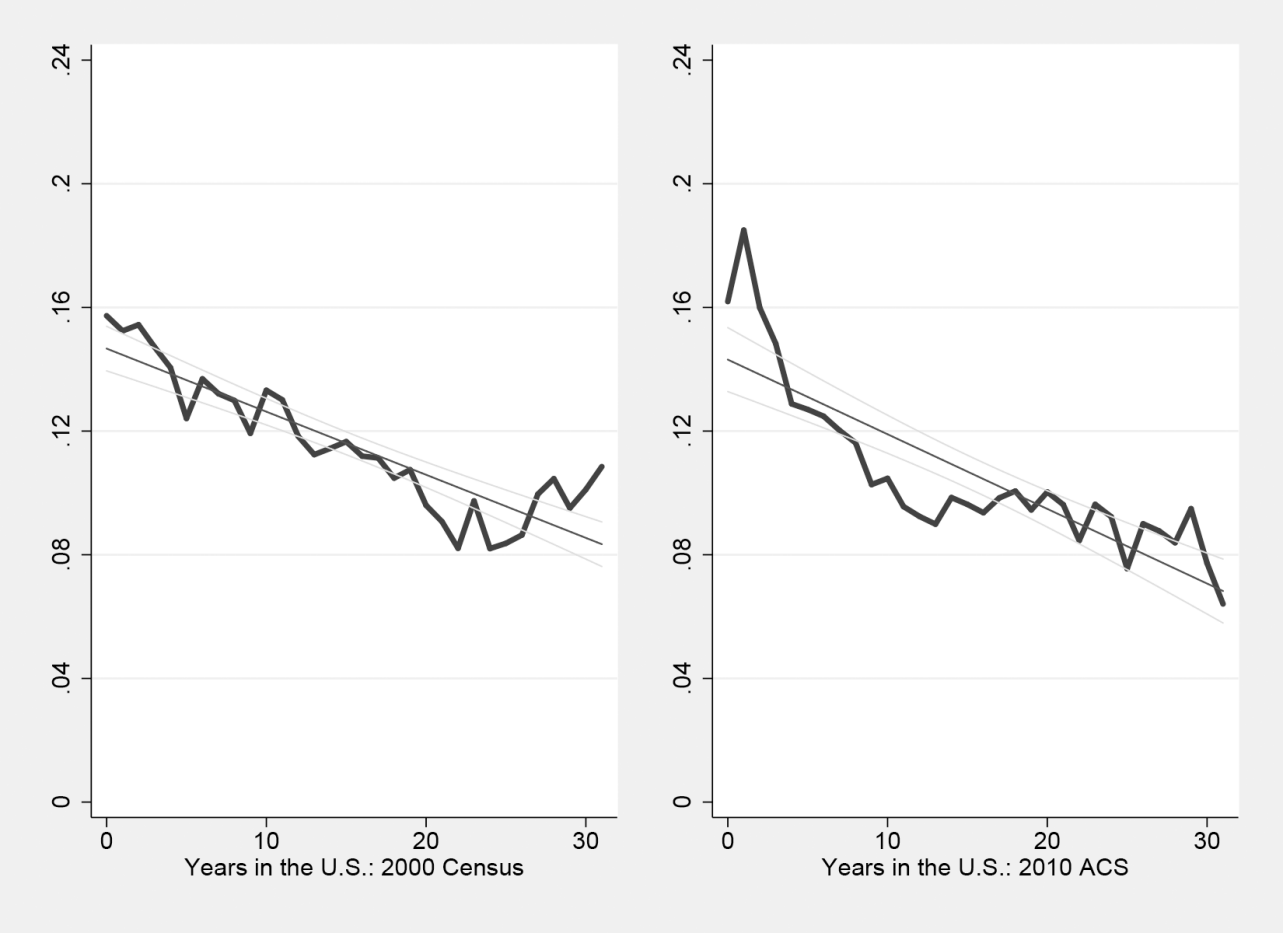
Public transit rate and the length of stay: After 2000.

In the following Figs 1–3, I descriptively present the public transit rate among U.S. immigrants as the function of their length of stay. In each figure I also show the simple linear regression line with the 95% confidence interval. Fig 1 focuses on the full sample: in general, 20% of immigrants use public transit upon arrival; the public transit rate dramatically decreases in the first few years after arrival, then slowly decreases and converges to 10%, which is still much higher than that among natives.

Figs 2 and 3 show the public transit rate as the function of length of stay based on each sub-sample. Graphically, the linear regression line appears to be relatively steeper in the 1980 and 1990 sample. This could suggest that the rate of transportation assimilation decreases over time. Note that, however, the “starting point” among earlier cohorts of immigrants surveyed in 1980 and 1990 is also higher. In other words, the public transit rate among new immigrants upon arrival becomes relatively low in recent decades. This might partially account for the decrease in the rate of transportation assimilation.

Again, Fig 2 is partially affected by data limitations that only year intervals of the length of stay (rather than exact years) were asked in the 1980 and 1990 census, but the general shape and qualitative pattern of transportation assimilation shown in Fig 2 are very similar to those shown in Figs 1 and 3.

### Regression models

Consider an immigrant *i* originally from country *k* and live in county *c* in the U.S. The survey year is *j*, where *j* ∈ {1980, 1990, 2000, 2010}. Denote *P*_*ijkc*_ as the binary indicator of public transit use and I run a baseline linear probability model (LPM) to examine the rate of transportation assimilation:

$$\begin{array}{c} P_{ijkc}=\alpha_{0}+\alpha_{1}l_{ijkc}+X_{ijkc}\alpha_{2}+\tau_{j}+\tau_{k}+\tau_{jc}+\epsilon \end{array}$$ (1)

where *l*_*ijkc*_ is the length of stay in the U.S., and **X**_*ijkc*_ is the vector of regressors introduced earlier. *τ*_*j*_, *τ*_*k*_, and *τ*_*jc*_ are year fixed effects, country-of-origin fixed effects, and county-year fixed effects, respectively. *ϵ* is the error term. Including year fixed effects helps control for year-specific factors, such as that the public transit ridership rate among newcomers might be different in difference census years due to year-specific characteristics. Here, *α*_1_ is the rate of transportation assimilation.

With this specification, it is easy to establish similar logit or probit models. In the empirical analysis I will report odds ratios of logit regressions. While not presented in this paper, the marginal effects of logit models are numerically very similar to the OLS coefficients, and the results of this paper are not driven by the statistical models.

The above equation produces the general estimation. To examine transportation assimilation by year, I further run four regressions based on sub-samples that contain individuals taking the survey in specific years. In each regression, *τ*_*j*_ is canceled out and *τ*_*jc*_ is simplified as *τ*_*c*_. These regression models estimate the assimilation rate in each survey year, i.e., $\alpha_{1}^{1980}$, $\alpha_{1}^{1990}$, $\alpha_{1}^{2000}$, and $\alpha_{1}^{2010}$.

Table 1 suggests the rate of public transit ridership among U.S. immigrants has steadily fallen since 1980. Has the rate of transportation assimilation also changed during the past decades? To examine the trend in transportation assimilation, I further pool the data set and employ the fixed effect models to compare coefficients of length of stay *l*_*i*_ by census year. By observing the regression results of these models one can check whether the decreasing trend of transportation assimilation is statistically significant.

## Results

I now report the empirical results of this paper. In Table 2, I report odds ratios of simple logit models, in which I regress public transit ridership only on the length of stay in the U.S. using the immigrant sample. Column 1 presents evidence of transportation assimilation: public transit ridership decreases as the length of residency in the U.S. increases. Logit models in Column 2, 3, 4, and 5 further show that the rate of transportation assimilation could change over time: the assimilation rate appears to be relatively lower after 2000.

**Table 2:** Transportation assimilation: The immigrant sample.

	(1)	(2)	(3)	(4)	(5)
	Full	1980	1990	2000	2010
Length of stay	0.9780***	0.9773***	0.9713***	0.9788***	0.9804***
	[-7.49]	[-6.54]	[-6.24]	[-6.68]	[-7.54]
Constant	0.2420***	0.2438***	0.2110***	0.1772***	0.1557***
	[-6.59]	[-6.20]	[-9.04]	[-8.97]	[-9.89]
Year FE	Yes	—	—	—	—
Other controls	No	No	No	No	No
County-year FE	No	No	No	No	No
Pseudo R^2^	0.014	0.007	0.013	0.009	0.008
Observations	537,566	62,934	106,740	167,443	200,449

Logistic regression odds ratio are reported.

*: *p* <.05.

**: *p* <.01.

***: *p* <.001.

*z*-statistics are in brackets, and are clustered at the county-year level.

In Table 3 I rerun the five regressions, but now including demographic and socioeconomic variables. Results shows the same qualitative pattern: transportation assimilation does occur among immigrants; moreover, the assimilation rate decreases over time. Compared with findings of this table, the assimilation rate is underestimated in Table 2 where demographic and socioeconomic characteristics are included. Table 3 shows that female immigrants and older immigrants are more likely to commuter by public transit, while married immigrants and immigrants with higher income are less likely to ride public transit, and the effect of education appears to be minor. The number of children is negatively correlated with public transit ridership, and the place of work indeed has great effects on travel behaviors, in the sense that those working in central metropolitan areas are much more likely to take public transit, and those working outside metropolitan areas are less likely to be public transit riders.

**Table 3:** Transportation assimilation: The immigrant sample.

	(1)	(2)	(3)	(4)	(5)
	Full	1980	1990	2000	2010
Length of stay	0.9824***	0.9762***	0.9748***	0.9840***	0.9867***
	[-12.47]	[-8.84]	[-8.21]	[-9.27]	[-10.36]
Female	1.2311***	1.2430**	1.3583***	1.2477***	1.1613***
	[10.44]	[3.24]	[5.84]	[7.10]	[7.28]
Age	1.0072**	1.0229**	0.9998	1.0095*	0.9971
	[2.65]	[3.11]	[-0.03]	[2.22]	[-0.69]
Age^2^	1.0001***	1.0000	1.0000	1.0000	1.0001**
	[2.02]	[-0.31]	[0.16]	[0.23]	[2.85]
Working in central metropolitan areas	6.2941***	1.9398***	5.7792***	8.4003***	9.4598***
	[10.32]	[9.19]	[7.75]	[10.68]	[9.94]
Working outside metropolitan areas	0.3387***	0.1548***	0.2518***	0.4213***	0.4348*
	[-4.84]	[-6.96]	[-6.96]	[-3.57]	[-2.52]
Hispanic, white	1.0713	1.4966*	1.0703	1.0438	1.0397
	[0.63]	[2.48]	[0.57]	[0.41]	[0.34]
Hispanic, others	1.3175*	1.3548*	1.2130	1.1741	1.3886*
	[2.40]	[2.33]	[1.36]	[1.51]	[2.59]
Black	1.6906***	2.3054***	2.0315***	1.5274***	1.4375**
	[4.26]	[4.00]	[6.19]	[4.01]	[3.40]
Asian	1.3145***	1.2476*	1.3726**	1.2608***	1.3680***
	[5.83]	[2.55]	[3.20]	[4.59]	[3.52]
Married	0.6451***	0.6929***	0.6338***	0.6370***	0.6340***
	[-18.15]	[-8.89]	[-11.84]	[-12.09]	[-16.56]
Number of children	0.8936***	0.8997***	0.9090***	0.8959***	0.8763***
	[9.46]	[-6.41]	[-4.58]	[-10.05]	[-10.87]
Years of schooling	0.9710***	0.9791**	0.9694***	0.9659***	0.9742***
	[-7.31]	[-3.32]	[-6.08]	[-6.44]	[-7.17]
Log(personal income)	0.9752***	0.9747**	0.9766**	0.9717***	1.0141
	[-3.90]	[-3.40]	[-2.80]	[-5.74]	[0.54]
Constant	0.0831***	0.0074***	0.0986	0.0446***	0.0489**
	[-3.69]	[-13.06]	[-1.52]	[-3.93]	[-3.22]
Year FE	Yes	—	—	—	—
Occupation FE	Yes	Yes	Yes	Yes	Yes
Other controls	Yes	Yes	Yes	Yes	Yes
County-year FE	No	No	No	No	No
Pseudo R^2^	0.217	0.168	0.227	0.235	0.248
Observations	537,339	62,323	106,081	166,382	199,335

Logistic regression odds ratio are reported.

*: *p* <.05.

**: *p* <.01.

***: *p* <.001.

*z*-statistics are in brackets, and are clustered at the county-year level.

In Table 4 I add another important determinant of transportation mode choices in the models: the geographic factors. Table 3 has presented the positive effect of working in central metropolitan areas and the negative effect of working outside metropolitan areas on public transit use. Table 4 further takes county information into account, in order to control for the possibility that different counties provide different levels of public transit services for travelers. Compared with Tables 3 and 4 shows that the assimilation rate is indeed underestimated without controlling for the country of residence. This suggests that the uneven distribution of public transit systems in the U.S. could interact with immigrants’ settlement patterns and further the rate of transportation assimilation. Converting the coefficients into the “effect” of the length of stay in the U.S., the models do predict that the likelihood of public transit ridership after immigrants stay as long as the average length of stay in the U.S. is approximately 10% (taking the public transit rate upon arrival into account) in each sub-sample, which is very close to that presented in Fig 2.

**Table 4:** Transportation assimilation: The immigrant sample.

	(1)	(2)	(3)	(4)	(5)
	Full	1980	1990	2000	2010
Length of stay	0.9814***	0.9756***	0.9732***	0.9830***	0.9856***
	[-8.52]	[-7.51]	[-6.86]	[-7.15]	[-7.18]
Female	1.4036***	1.5953***	1.5375***	1.4053***	1.2788***
	[15.28]	[11.36]	[9.31]	[9.39]	[7.95]
Age	0.9922**	0.9986	0.9921	0.9922	0.9832
	[-2.80]	[-0.20]	[-1.20]	[-1.88]	[-3.56]
Age^2^	1.0002***	1.0002**	1.0003***	1.0002***	1.0003***
	[7.11]	[2.74]	[3.80]	[4.09]	[4.79]
Working in central metropolitan areas	3.2968***	1.5731***	3.9974***	5.2627***	5.8501***
	[9.12]	[6.04]	[8.99]	[12.52]	[11.84]
Working outside metropolitan areas	0.5097***	0.2759***	0.3887***	0.9098	0.7230
	[-3.61]	[-5.27]	[-4.55]	[-0.59]	[-1.17]
Hispanic, white	1.0314	1.2855*	1.0097	0.9782	1.0594
	[0.62]	[2.29]	[0.14]	[-0.34]	[0.78]
Hispanic, others	1.1353*	1.2378	1.1048	1.0056	1.1894**
	[2.30]	[1.86]	[1.17]	[0.08]	[2.74]
Black	1.5171***	1.7409***	2.0315***	1.4197***	1.4677***
	[6.17]	[4.85]	[4.50]	[5.37]	[4.96]
Asian	1.1167**	1.0922	1.0563	1.0997	1.1375
	[3.32]	[0.95]	[0.66]	[1.55]	[1.17]
Married	0.6882***	0.7406***	0.6462***	0.5805***	0.6933***
	[-12.44]	[-7.93]	[-9.34]	[-9.32]	[-13.24]
Number of children	0.8999***	0.9139***	0.9131***	0.9023***	0.8750***
	[-14.97]	[-6.04]	[-5.27]	[-10.88]	[-15.34]
Years of schooling	0.9806***	0.9974	0.9760***	0.9741***	0.9842***
	[-4.06]	[-0.36]	[-6.08]	[-5.88]	[-3.49]
Log(personal income)	0.9612***	0.9552***	0.9643***	0.9638***	0.9408**
	[-6.56]	[-5.97]	[-4.46]	[-6.63]	[-2.87]
Constant	0.0109***	0.0075***	0.0986	0.0027***	0.0133**
	[-11.61]	[-15.05]	[-1.52]	[-10.20]	[-6.97]
Year FE	Yes	—	—	—	—
Occupation FE	Yes	Yes	Yes	Yes	Yes
Other controls	Yes	Yes	Yes	Yes	Yes
County-year FE	Yes	Yes	Yes	Yes	Yes
Pseudo R^2^	0.319	0.305	0.320	0.317	0.347
Observations	528,389	59,457	101,512	158,772	191,849

Logistic regression odds ratio are reported.

*: *p* <.05.

**: *p* <.01.

***: *p* <.001.

*z*-statistics are in brackets, and are clustered at the county-year level.

Previous tables show some evidence that the rate of transportation assimilation has decreased in the past few decades. To further examine this, In Table 5 I pool the data and construct variables that present cross-sectional differences in the assimilation rate. To do so, I conduct pairwise comparisons of coefficients of length of stay between two sub-samples. In Column 1 I run the regression of transportation assimilation based on the 1980 and 1990 sample. Results show that the assimilation rate estimated in the 1990 sample is not significantly different from that estimated in the 1980 sample. Column 2 suggests that the assimilation rate has steadily decreased in the 1990s. Similarly, Column 3 shows that immigrants of the 2010 cohort could assimilate faster than those of the 2000 cohort. In Column 4 I split the full sample by year 2000 (as there are four sub-samples in total). This final regression shows that the rate of transportation assimilation among U.S. immigrants is indeed significantly smaller in the 2000 and 2010 (i.e., “post-2000” in Table 5) sample.

**Table 5:** Transportation assimilation: Pairwise coefficient comparison.

	(1)	(2)	(3)	(4)
	1980 & 1990	1990 & 2000	2000 & 2010	Full
Length of stay	0.9737***	0.9759***	0.9804***	0.9787***
	[-6.69]	[-20.99]	[-24.73]	[-23.78]
(Length of stay)*	0.9967			
1990 dummy	[-1.49]			
(Length of stay)*		1.0050***		
2000 dummy		[3.58]		
(Length of stay)*			1.0025*	
2010 dummy			[2.22]	
(Length of stay)*				1.0030**
post-2000 dummy				[2.90]
Year FE	Yes	Yes	Yes	Yes
Occupation FE	Yes	Yes	Yes	Yes
Other controls	Yes	Yes	Yes	Yes
County-year FE	Yes	Yes	Yes	Yes
Pseudo R^2^	0.309	0.272	0.322	0.282
Observations	164,538	265,236	357,662	528,389

Logistic regression odds ratio are reported.

*: *p* <.05.

**: *p* <.01.

***: *p* <.001.

*z*-statistics are in brackets, and are clustered at the county-year level.

Note that results in Table 5—regarding trends in the rate of transportation assimilation over time—need to be interpreted with caution. The “starting point” of transportation assimilation appears to be different in each census year. In particular, not only the starting point of immigrants’ transportation behaviors changes over time, which is largely due to changes in immigrants’ characteristics, the starting point of transportation behaviors among natives could also change over time. Hence, one caveat of Table 5’s analysis is that it only shows trends in the “absolute” rate of transportation assimilation, which measures how immigrants follow natives’ social norms to travel less by public transit; however, it might not necessarily indicate trends in immigrants’ transportation behaviors relative to natives. This is a general statistical issue for individual-level datasets, as it is difficult to identify comparable individuals in the control group (i.e., natives) for every individual in the immigrant sample.

## Discussion

Consistent with earlier findings [13], the results of this paper suggest transportation assimilation among immigrants in the U.S., i.e., immigrants are much more likely to ride public transit upon arrival, but then rely less on public transit as the length of stay in the U.S. increases. Based on repeated cross-sectional data, this paper further contributes to the literature by pointing out the rate of transportation assimilation has decreased over time. This section first discusses possible mechanisms behind this phenomenon, and then discusses policy implications and limitations of the empirical analysis.

### Mechanisms

There are several possible reasons that could explain this phenomenon. Figs 1 and 2 suggest that the “starting point” of transportation assimilation might change over time. Indeed, approximately 20% of immigrants were public transit users upon arrival in 1980 and 1990, and then this rate decreased afterwards (especially in 2000). This, however, could not fully explain the findings of this paper. On one hand, year fixed effects could partially control for this factor. On the other hand, this can be analyzed by running regressions of transportation assimilation based on immigrants who have just arrived in the U.S., say, within 10 years. Table 6 presents results of these regressions. Results of Column 3 and 4 suggest that the assimilation rate actually appears to be even slightly larger among new immigrants in 2000 and 2010.

**Table 6:** Transportation assimilation: Length of stay less than 10 years.

	(1)	(2)	(3)	(4)
	Full	Full	1980 & 1990	2000 & 2010
Length of stay	0.9513***	0.9397***	0.9432***	0.9332***
	[-9.37]	[-15.66]	[-12.20]	[-10.89]
Constant	0.0823***	0.0055***	0.0013***	0.0042***
	[-3.09]	[-9.14]	[-5.96]	[-8.05]
Year FE	Yes	—	—	—
Occupation FE	Yes	Yes	Yes	Yes
Other controls	Yes	Yes	Yes	Yes
County-year FE	No	Yes	Yes	Yes
Pseudo R^2^	0.177	0.285	0.281	0.299
Observations	162,839	157,940	61,438	93,246

Logistic regression odds ratio are reported.

*: *p* <.05.

**: *p* <.01.

***: *p* <.001.

*z*-statistics are in brackets, and are clustered at the county-year level.

Second, the U.S. has greatly updated its public transit systems in past decades, especially in many urban areas in the Midwest and West. Hence, different cohorts of immigrants are exposed to different public transit systems upon arrival, which could affect the rate of transportation assimilation. This, however, has been captured in the regression models by controlling for geographic factors such as county and the location of workplace. This could be seen from the coefficients of workplace locations in Tables 3 and 4, as well as the change in the assimilation rate estimated in Tables 3 and 4, before and after county fixed effects are included.

Third, older surveys are relatively “biased” towards immigrants who have fewer years of stay in the U.S. Table 1 suggests the average years of stay have largely increased over the past decades, and immigrants’ assimilation might not be linear. Indeed, immigrants does not socially or economically assimilate into the U.S. at a constant rate [7]. Table 6 also suggests that newcomers generally assimilate faster. Hence, I extend the statistical model by introducing a quadratic term of the length of stay in Table 7.

**Table 7:** Transportation assimilation: The quadratic term.

	(1)	(2)	(3)	(4)	(5)
	Full	1980	1990	2000	2010
Length of stay	0.9685***	0.9528***	0.9499***	0.9799***	0.9695***
	[-8.67]	[-7.24]	[-6.50]	[-6.61]	[-8.57]
Length of stay squared	1.0003***	1.0008***	1.0007***	1.0003***	1.0003***
	[6.29]	[4.80]	[4.88]	[4.20]	[5.93]
Constant	0.0104***	0.0073***	0.0117**	0.0026***	0.0124***
	[-11.64]	[-15.30]	[-3.06]	[-10.28]	[-7.08]
Year FE	Yes	—	—	—	—
Occupation FE	Yes	Yes	Yes	Yes	Yes
Other controls	Yes	Yes	Yes	Yes	Yes
County-year FE	Yes	Yes	Yes	Yes	Yes
R^2^	0.319	0.306	0.321	0.314	0.348
Observations	528,389	59,457	101,512	158,772	191,849

Logistic regression odds ratio are reported.

*: *p* <.05.

**: *p* <.01.

***: *p* <.001.

*z*-statistics are in brackets, and are clustered at the county-year level.

Results show that the coefficient of this quadratic term is positive and statistically significant. Hence, the rate of transportation assimilation should be higher in a sample that contains more new immigrants. As the average length of stay has steadily increased (reported in Table 1), many immigrants in recent surveys have stayed long enough to assimilate into the U.S. transportation pattern. Hence, Tables 6 and 7 suggest that the decreasing trend of transportation assimilation over time can be explained by the changing demographics of U.S. immigrants, in the sense that the average length stay in the U.S. among immigrants has significantly increased.

Another factor of the demographics of U.S. immigrants is the racial composition of U.S. immigrants in different decades. In the context of this paper, The number of white immigrants—especially non-Hispanic whites—has greatly decreased over the past decades. The proportion of Hispanic immigrants has fluctuated, as seen in Table 1 and related research [28], but the Asian population in the U.S. has steadily increased. Compared with non-Hispanic white immigrants (in Table 3), Hispanic immigrants are generally not significantly different but Asian immigrants are significantly likely to take public transit. Hence, the trend of transportation assimilation might be affected by different demographic backgrounds of immigrants of different cohorts. Table 8 focuses on transportation assimilation using the Hispanic and Asian sample in two panels.

**Table 8:** Transportation assimilation: Hispanic and Asian immigrants.

	(1)	(2)	(3)	(4)	(5)
	Full	1980	1990	2000	2010
**Hispanic**:					
Length of stay	0.9778***	0.9730***	0.9697***	0.9804***	0.9827***
	[-5.96]	[-3.79]	[-5.52]	[-5.99]	[-4.34]
Constant	0.0207***	0.0587*	0.0145***	0.0051***	0.0316***
	[-9.75]	[-2.19]	[-3.94]	[3.97]	[-4.11]
Pseudo R^2^	0.298	0.307	0.290	0.280	0.333
Observations	217,131	18,292	41,371	66,157	80,174
**Asian**:					
Length of stay	0.9828***	0.9836*	0.9681***	0.9847***	0.9873***
	[-5.09]	[-2.37]	[-6.53]	[-3.77]	[-3.66]
Constant	0.0168***	0.0040***	0.0414**	0.2481	0.1826*
	[-8.56]	[-7.57]	[-3.37]	[-1.68]	[-2.09]
Pseudo R^2^	0.321	0.272	0.341	0.336	0.352
Observations	120,325	9,088	20,955	33,940	47,911
All controls	Yes	Yes	Yes	Yes	Yes

Logistic regression odds ratio are reported.

*: *p* <.05.

**: *p* <.01.

***: *p* <.001.

*z*-statistics are in brackets, and are clustered at the county-year level.

Table 8 shows that in the full sample as well as sub-samples of specific survey years, Asian immigrants appear to assimilate at slower rates in terms of public transit ridership. In other words, many Asian immigrants might still take public transit even after staying in the U.S. for many years. While not reported here, I find similar results of transportation assimilation among Asian immigrants and other immigrants upon arrival. As the Asian population has steadily grown in the U.S., the changing racial composition in the U.S. could be another factor behind the trend of transportation assimilation in the past decades.

To further study this, I compare the Hispanic and Asian immigrants in Table 9. Two immigrant populations have similar average years of stay and, among newcomers who have stayed in the U.S. no more than one year, the rate of public transit ridership appears to be very similar. There are at least two possible reasons behind how two the Asian and Hispanic population assimilate differently afterwards. First, transporting children is an important reason of driving, and Hispanic immigrants generally have more children. Second, settlement patterns between Hispanic and Asian immigrants are substantially different. In particular, Asian immigrants are much more likely to work in central metropolitan areas (where taking public transit is very common) and less likely to work outside metropolitan areas (where public transit systems are inconvenient or even unavailable). In the last row of Table 9 I rerun the regressions reported in Column 1, Table 8, without controlling for county fixed effects. Results show that the comparative pattern of transportation assimilation between two immigrant populations actually reverses without taking county of residence into account. Hence, the geographic distribution of workplace and residence might explain the pattern of transportation assimilation among Asian immigrants.

**Table 9:** Comparison: Hispanic and Asian immigrants in the sample.

	Hispanic		Asian	
% in 1980 & 1990^†^	0.382	(0.486)	0.207	(0.405)
% in 2000 & 2010^†^	0.433	(0.496)	0.252	(0.434)
Length of stay	16.060	(11.612)	15.247	(10.647)
Public transit, newcomers	0.185	(0.389)	0.184	(0.387)
Number of children	1.252	(1.406)	1.053	(1.195)
Central metropolitan areas	0.210	(0.407)	0.273	(0.445)
Outside metropolitan areas	0.059	(0.235)	0.037	(0.189)
Odds ratio, no county FE^‡^	0.9856	[-5.82]	0.9834	[-6.64]
Observations	224,252		127,669	

†: Proportion of Hispanic/Asian immigrants in the immigrant sample.

‡: Replication of regressions in Column 1, Table 8, but without county FE.

Standard deviations are in parentheses. ‡: *z*-statistics are reported.

### Policy implications

This section analyzes possible mechanisms behind transportation assimilation. There are several factors that affect transportation behaviors among both natives and immigrants, such as the availability of public transit. However, there exist some other determinants that are more specific to immigrants. The main empirical analysis in Tables 3 and 4 points out the importance to control for immigrants’ individual characteristics such as education, occupation, income, and area of residence. Demographic characteristics such as race and ethnicity also play an important role. These have been well documented in many previous studies [4, 5, 24–27].

This section further points out that it is important to focus on the intersection between individual socioeconomic and demographic characteristics, which is especially helpful for policy makers: immigrants are highly segregated [2], and each immigrant population defined by race and ethnicity, if considered as a unity, usually has particular locational choices and settlement patterns [6, 7, 21]. For example, as reflected in Table 9, while Asian immigrants are generally considered to be socioeconomically very assimilated [8, 12], they usually reside in central metropolitan areas with good public transit services. Hence, transportation assimilation among Asian immigrants appears to be inconsistent with their socioeconomic assimilation, especially compared with Hispanic immigrants.

For policy makers, such a population-based analytical framework might be more useful than the interpretation of determinants of transportation assimilation at the individual level. In particular, many services—such as language signs and updates of public transit for ethnic enclaves [2]—are specific for populations by race and ethnicity. The results of this section highlight the importance of focusing on “gateway cities” for specific immigrant populations when making transportation policies, as the rate of transportation assimilation is highly associated with geographic locations.

### Limitations

To conclude this section, I finally discuss some limitations of the statistical analysis. First, both the census and ACS only ask questions about commuting modes, hence transportation tools for other purposes, such as shopping, are not surveyed. Second, because I cannot link individuals across samples, cross-sectional results might be driven by biases from departures of return immigrants. This is a major statistical issue in studies of economic history [34–36], although the rate of return migration is relatively low in recent decades. Note that although immigrants answer survey questions based on the county of residence, the census and ACS also survey some questions about the place of work, and prior research has also exploits variables related to residential and workplace locations [37] to study immigrants’ commuting behaviors.

## Conclusion

This paper studies a question in urban geography: transportation assimilation among U.S. immigrants. Specifically, new immigrants in the U.S. are more likely to take public transit and less likely to drive to commute. Prior research, however, points out that immigrants start to ride public transit less as the length of stay increases.

In this paper, I use repeated cross-sectional data and revisits this empirical question. The major advantage of using repeated cross-sectional data is that it is now possible to analyze how the rate of transportation assimilation changes over time, and what factors could affect the trend of transportation assimilation.

Consistent with prior research, this paper finds evidence of transportation assimilation: as the length of stay in the U.S. increases, immigrants are less likely to take public transit. Moreover, this paper also finds that the assimilation rate has decreased over time, despite the fact that public transit ridership among immigrants has also decreased. There are several factors behind the overall trend of transportation assimilation in the past few decades. First, new immigrants of recent cohorts become slightly less likely to take public transit upon arrival. Second, geographic variables related to residence and workplace play an important role in affecting immigrants’ public transit ridership, which means that immigrants’ changing settlement patterns and recent development of public transit system could be determinants of transportation assimilation. Finally, the changing demographics of U.S. immigrants have significant effects on the trend of transportation assimilation. Specifically, the average length of stay in the U.S. has steadily increased in the past decades, and the proportion of Asian immigrants—many of whom work in central metropolitan areas—has increased. These factors also lead to the lower rate of transportation assimilation.
